# Inpatient generalist palliative care during the SARS-CoV-2 pandemic – experiences, challenges and potential solutions from the perspective of health care workers

**DOI:** 10.1186/s12904-022-00958-9

**Published:** 2022-05-03

**Authors:** Manuela Schallenburger, Marie Christine Reuters, Jacqueline Schwartz, Marius Fischer, Carmen Roch, Liane Werner, Claudia Bausewein, Steffen T. Simon, Birgitt van Oorschot, Martin Neukirchen

**Affiliations:** 1 Interdisciplinary Centre for Palliative Medicine, University Hospital, Heinrich Heine University, Moorenstraße 5, 40225 Duesseldorf, Germany; 2 Interdisciplinary Centre for Palliative Medicine, University Hospital Wuerzburg, Wuerzburg, Germany; 3 Department of Palliative Medicine, LMU University Hospital, Munich, Germany; 4 Department of Palliative Medicine and Center for Integrated Oncology Aachen Bonn Cologne Dusseldorf (CIO ABCD), University of Cologne, Faculty of Medicine and University Hospital, Cologne, Germany; 5 Department of Anesthesiology, University Hospital, Heinrich Heine University, Duesseldorf, Germany

**Keywords:** Inpatient generalist palliative care, Visiting regulation, Farewell, Communication, Pandemic, Relatives

## Abstract

**Background:**

The SARS-CoV-2 pandemic has presented major challenges to the health system. Despite high acute case numbers, patients without Covid-19 still need to be cared for. Due to the severity of the disease and a possible stressful overall situation, patients with palliative care needs also require comprehensive care during pandemic times. In addition to specialized palliative care facilities, this also takes place in non palliative care wards. In order to ensure this general palliative care also in pandemic times, the experience of the staff should be used. The aim of this paper is to examine challenges and possible solutions for general palliative care inpatients in relation to the care of seriously ill and dying patients and their relatives.

**Methods:**

Qualitative semi-structured focus groups were conducted online for the study. Participants were staff from intensive care or isolation wards or from units where vulnerable patients (e.g. with cognitive impairment) are cared for. The focus groups were recorded and subsequently transcribed. The data material was analysed with the content structuring content analysis according to Kuckartz.

**Results:**

Five focus groups with four to eight health care professionals with various backgrounds were conducted. Fifteen main categories with two to eight subcategories were identified. Based on frequency and the importance expressed by the focus groups, six categories were extracted as central aspects: visiting regulations, communication with relatives, hygiene measures, cooperation, determination of the patients will and the possibility to say good bye.

**Conclusion:**

The pandemic situation produced several challenges needing specific solutions in order to manage the care of seriously ill and dying patients. Especially visiting needs regulation to prevent social isolation and dying alone. Finding alternative communication ways as well as interprofessional and interdisciplinary cooperation is a precondition for individualised care of seriously ill and dying patients and their relatives. Measures preventing infections should be transparently communicated in hospitals.

## Introduction

The SARS-CoV-2 pandemic poses new challenges for all areas of healthcare systems. So far, the focus has mostly been on the establishment of intensive care beds and mechanical ventilation stations for patients acutely ill with COVID-19 (coronavirus disease).

Even though Germany was not as affected as other European countries or the USA during the first wave (March–May 2020) with 182,135 COVID-19 cases and a peak of 2,928 patients in the intensive care unit in one day, people became severely ill with COVID-19 and died despite intensive care therapy [1, 2]. At the same time, worse care of patients without SARS-CoV-2 was described [3]. On the one hand, patients avoided physician and hospital visits in general [4]. On the other hand, surgeries, interventions, and therapies had to be postponed due to the prioritized care of patients with COVID-19. To maintain good care for seriously ill and dying patients during the pandemic, the German Association for Palliative Medicine published early recommendations for patients with COVID-19 as well as recommendations for prehospital decision making and prioritization of patients when resources are scarce [5, 6]. Within discussions in our regular meetings with the research consortium "National Strategy for Palliative Care in Pandemic Times (PallPan)" reports from patients, primary care physicians, nursing homes and palliative care providers were gathered, which expressed that both, the identification of individual treatment preferences, and the quality of care for seriously ill and dying patients varied widely. Lack of protective clothing as well as strict isolation measures and visiting bans led to great distress among those affected. At the same time, creative ideas emerged with the aim of maintaining palliative care for patients and relatives under the prevailing contact restrictions.

Overall, it became clear that the German healthcare system was not sufficiently prepared for the challenges of palliative care in pandemic times. To be better prepared in the future, national recommendations and concepts are needed that focus on generalist (i.e. a basic skillset of palliative care that all physicians should have [7]) as well as specialized outpatient and inpatient palliative care. This should ensure that "pandemic preparedness" can also be achieved for the care of seriously ill and dying [8].

Against this background, twelve university palliative medicine centers, have joined forces in the Network University Medicine (NUM), funded by the Federal Ministry of Education and Research, to form the Palliative Medicine Research Network. The project PallPan aims to explore the challenges and solutions concerning the care for seriously ill, dying and deceased patients (with/without COVID-19) and their relatives or bereaved in the different areas of palliative care, to provide consensual recommendations for action for adequate care of dying patients and their relatives in pandemic times. The study group is hereafter referred to as the PallPan consortium.

This study describes the experiences, challenges and solutions in inpatient generalist palliative care in hospitals of regular, priority and maximum care. Primary care hospitals offer at least service in surgery and internal medicine. Secondary care hospitals also include further specialties, f.i. gynecology and obstetrics, ophthalmology and orthopedics. Tertiary care hospitals offer service in nearly all existing medical disciplines.

The results are incorporated directly into the recommendations for action of the PallPan consortium [9].

### Palliative care in Germany

The palliative care system in Germany can be divided in inpatient and outpatient as well as in generalist and specialized palliative care.

Outpatient generalist palliative care is provided mainly by general practitioners and nursing services. Outpatient specialized palliative care is initiated in case of complex symptom burden and is provided by palliative care physicians and nurses at home, in nursing homes or in hospices.

Inpatient generalist palliative care is provided for all hospitalized patients suffering from life threatening diseases, while specialized palliative care service is provided on palliative care wards or within specialized service on regular or intensive care wards.

## Methods

### Design

This is a qualitative study with multiprofessional focus groups. Reporting follows the COREQ guidelines [10].

### Setting and participants

Inclusion criteria were working as a physician, nurse, or member of another profession in an

- intensive care unit,
- isolation ward or
- ward with particularly (especially cognitively) burdened patients (e.g. neurology and geriatrics).

Exclusion criterion was training in specialized palliative care.

Convenience sampling was used to select participants.

Health Care workers from all hospital categories (primary, secondary, tertiary care) were chosen because all of them treat a large number of seriously ill and dying patients (with/without COVID-19). Participants were expected to have worked on the same ward since at least March 2020. This rather heterogeneous composition of the groups was chosen as it resembles the large spectrum of generalist palliative care.

### Recruitment and data collection

Participants were approached by email through members of the PallPan consortium. The participants themselves were not part of the consortium, which reduces the risk of selection bias. They were informed in writing in advance and verbally during the focus group interview about the aim of the study and the role of the facilitators in the project. There were no direct or hierarchical relationships between the moderator/author team and the participants. Due to applicable contact restrictions, the focus group interviews took place online using the Cisco Webex videoconferencing tool.

The semi-structured focus groups aimed to elicit participants' experiences, challenges, and potential solutions during the first wave (March–May 2020). The interview guide consisted of the aforementioned three thematic blocks, each of which was opened by a general introductory question.

1. What did your everyday professional life look like during the last 6 months since the beginning of the SARS-CoV-2 pandemic regarding severly ill and dying patients, and their relatives, also in comparison to pre-pandemic times? Which experiences did you have, think about the different phases of lockdown and non lockdown periods?
2. Which challenges did you face during the last 6 months since the beginning of the SARS-CoV-2 pandemic? To what extent did these challenges differ in comparison to pre-pandemic times?
3. Which solutions were developed in your facility/institution/ward to overcome these challenges? Would you have wished for more support or solutions and from whom and what specifically?

Sub-questions related to the topic blocks and groups were subdivided into general and specific follow-up questions. The general questions, which were intended to maintain the conversation, included questions about the participants' daily work during the pandemic and what role seriously ill patients and their relatives took. Specific follow-up questions were intended to ask about content that may not have been named by the participants, but could be necessary for a closer understanding of their situation. Here, specific questions should be asked about e.g. therapy goals, support, workload, communication. These questions were developed within the interprofessional study team and were not pilot tested due to the time pressure during the pandemic. Nevertheless, they were checked for comprehension in the multi-professional study group. The follow-up questions were only used if the conversation between the participants got stuck or specific aspects remained open. However, due to the lively interactive exchange within the focus groups, they rarely had to be used. All interviews were facilitated by two moderators, both of whom were part of the study team. MCR, research assistant who was particularly employed for the study, acted as moderator and kept the interviews lively by asking follow-up questions when necessary. The co-moderator MS, research assistant having extensive experience in qualitative research especially the method of focus group interviews, had the task of documenting field notes and comprehension follow-up questions. All interviews were video recorded. The field notes or transcripts should capture the situation of the interview in order to consolidate perceptions in the text and the analysis. They were intended to provide support when needed because the video recordings were deleted after their transcription for reasons of data protection.

The number of interviews was previously set and recruitment was stopped as soon as the pre-defined participant numbers of the desired specialized field per focus group were fully stretched. According to the study protocol we planned five focus groups. One focus group each for ICU and isolation ward staff of tertiary and one focus group each for ICU and isolation ward staff in non-tertiary care hospitals. The fifth focus group was for participants, who work on wards for patients with special burden, without further differentiation of hospitals level of care. Five to ten participants were planned per focus group. Altogehter the minimum number of participants was 25.

### Data analysis

The interviews were transcribed according to the rules of Dresing and Pehl (emphasized words are thereby written in capital letters [11]). These transcripts formed the basis for the analysis of the data. The qualitative content analysis according to Kuckartz was used as the analysis method, which structures, analyzes, and interprets the data on the basis of deductively and inductively formed main and subcategories in seven steps, as shown in Table 1 [12].

**Table 1:** Procedure: Seven steps – qualitative content analysis according to Kuckartz [12]

Qualitative content analysis
1) Initiating text work
2) Development of thematic main categories
3) Coding of all material with those main categories
4) Compilation of all text passages with identically coded main categories
5) Inductive determination of subcategories on the material
6) Coding of the whole material with the evolved category system
7) Simple and complex analysis, visualization

After initiating text work, which included an initial reading of the transcripts, marking specifics and writing summaries, thematic main categories were developed. The material was then coded with these main categories and all text passages were compiled with the identically coded main categories. Afterwards subcategories were determined inductively to eventually code the whole material with the differiated category system. The last step was a simple and complex analysis and visualization.

Categories were formed deductively on the basis of the predefinded thematic blocks and inductively categories were developed based on the statements of the focus group participants, in order to identify challenges and solutions.

The analysis was initially conducted independently by each facilitator, while regularly checking each category against the transcripts and joint reflection. Subsequently, an exchange with further study authors took place to discuss the formed categories which were then interpreted and checked for possible connections. The weighting of key categories was based on the stated importance of the participants.

### Ethics vote and study registration

The research was performed in accordance with the Declaration of Helsinki. After registration in the German Register of Clinical Studies (DRKS00023595), the study was approved by the Ethics Committee of the Heinrich-Heine-University Duesseldorf (Reg. No. 2020–1119). All participants were informed before the start of the study and were given the opportunity for follow-up questions. All participants gave informed consent before being part of the study.

## Results

Members of the Pall Pan consortium identified 81 health care workers, that met the inclusion criteria from all over Germany. From those 81 possible participants 31 attended five focus groups, that took place between October 1^st^ to October 28^th^ 2020. The others refused participation due to work load during pandemic or scheduling difficulties. Each focus group comprised between four and eight participants and lasted 60–90 min. The reasons to refuse participation were primarily the high workload due to the high number of patients or not meeting the inclusion criteria and lacking replies. No further demographic data was collected despite profession, gender and type of hospital to be able to secure the identity of all participants. Furthermore, it was not relevant for achieving the study objective. In total, 19 doctors, nine nurses, one physiotherapist, one psychologist and one hospital hygeniest (a specialist trained in hygiene and infectiology, doctor or nurse, who fulfils tasks relating to advice and implementation of hygiene measures) took part in the interviews. The number of participating physicians is higher than those of other professions, especially compared to the nurses. The detailed allocation is shown in Table 2.

**Table 2:** Focus groups

Focus group 1	Focus group 2	Focus group 3	Focus group 4	Focus group 5
ICU	ICU	Isolation ward	Isolation ward	Provider of exceedingly burdened patients
maximum care	regular and priority care	maximum care	regular and priortiy	All hospitals
5 physicians	4 physicians	3 physicians	2 physicians	5 physicians
3 nurses	2 nurses	1 hospital hygienist	3 nurses	1 nurse
		1 physiotherapist		1 psychologist
Thereof 4 female	Thereof 6 male	Thereof 2 female	Thereof 4 female	Thereof 1 female

**Fig. 1:**
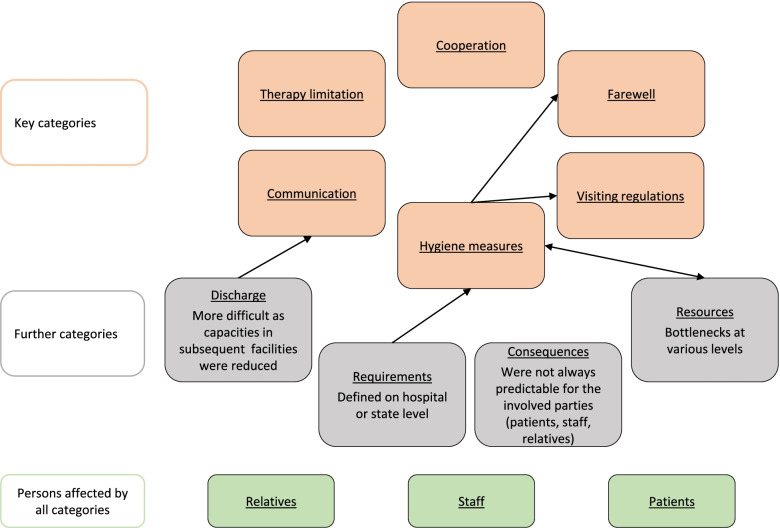
Categories

Although it was not intended, the evaluation showed data saturation. This was identified because there were hardly any new findings in the last interviews and no new findings in the last focus group conducted. In total, 13 main categories, having two to eight subcategories each, were extracted from the material. Furthermore, six key categories were identified. These together with the remaining main categories are shown in Fig. 1.

Table 3 shows the key categories and which content they comprise.

**Table 3:** Description of the key categories

Categories	Description
Visiting regulations	Comprises statements about prohibitions, restrictions, exceptions and rules with regards to visiting of patients
Communication with relatives	Comprises communication between staff and relatives, arising difficulties as well as possible alternative ways of communication
Hygiene measures	Comprises aspects of hygiene like the development and implementation of concepts and measures
Cooperation	Comprises cooperation on an interdisciplinary and interprofessional level and with other hospitals
Determination of the patients will	Comprises statements about reasons and circumstances limiting further medical treatment
Possibility to say farewell	Comprises remarks about the possibility to say farewell and to provide proper end-of-life care during pandemic times

### Visiting regulations

All participants named the existing visiting restrictions and prohibitions as a challenge for the patients. Due to banned visits, patients became lonely, leading to more work for the staff. So that they hardly knew how to do their work and which guidelines they should follow.

"However, [...] at the BEGINNING, when everyone was just so insecure, [...] there was this complete ban and then there was also no more left and right at first."

After some time, the participants reported, dying patients without SARS-CoV-2-Infection could be visited in exceptional cases. However, seriously ill, not immediately dying patients, already hospitalized for a long period, still could not be visited.

"So especially at the beginning [...], there were no visiting possibilities for not dying people at all, there are long-term intensive care patients over three, four weeks, who are simply NOT visited, [...] is [...] VERY, very difficult for the relatives."

For those infected with SARS-CoV-2, visiting restrictions were in force. At some hospitals, even up to the time of the interview, dying patients with COVID-19 often were not allowed to be visited. As the pandemic progressed, inconsistent handling of the given rules depending on the regional infection situation and the availability of protective equipment led to additional uncertainty and a feeling of injustice. Many of the participants reported that after the first wave of the pandemic had subsided, intensive efforts were made to find uniform solutions within the hospital. At most hospitals, one person per day was henceforth allowed to visit a patient at certain times, which was still not perceived as sufficient.

"Even the possibility to allow one relative for an hour per day is actually still not quite enough."

### Communication with relatives

The opportunities for staff to communicate with relatives were changing significantly as a result of the pandemic. Introduced visiting restrictions led to major communication deficits. For relatives it was rather difficult to comprehend the disease process without personal contact, because there was no visual impression.

"And relatives simply MISS the visual impression that is created in intensive care, what it means, that someone is critically ill."

Good and reliable communication with relatives was essential for the participants to include them in the course of events. Therapeutic interventions needed to be discussed with the authorized family members if the patient was unable to consent.

"This aspect of communication in the acute situation: How do I talk to the relatives?"

Nonface-to-face discussions about changes of goals of care pose an even greater challenge in terms of communication. Due to the pandemic-related restrictions, alternative ways of communication had to be identified, according to most participants.

"What was also very pronounced initially, but I also think is very important, is intensive contact via other ways, such as telephone calls."

Many participants mentioned that communication via (video) telephone was more difficult than in face-to-face meetings. Ensuring good communication with relatives in a critical situation without their physical presence was a considerable additional burden for the team, especially timewise.

"We did it in such a way that we made regular daily telephone calls, some with video, some without. Which was simply difficult to represent time-wise inpatient care on the side."

Weekly telephone consultations for relatives or conversations with particularly distressed relatives in the outside area of the clinics also provided alternative communication channels.

### Hygiene measures

From the beginning of the pandemic strict adherence to hygiene rules, which were infection control practices, was the focus of all participants efforts, despite the initial major shortage of protective equipment.

"We also had [...] no material. We started the [...] pandemic with seventy protective masks for a 430-bed hospital."

The implementation of hygiene measures with scarce resources resulted in a significant logistical and time overhead. Limited interaction due to the distance created and limited facial expressions due to the protective equipment, led to further challenges in dealing with each other for some participants.

"I found it very, very difficult (-) to maintain that DISTANCE."

Palliative care units were in some described cases converted to COVID-19 isolation wards, as these provided the best conditions for meeting hygiene standards. The participants were not part of the restructuring themselves, but experienced it because they could not transfer patients with palliative needs to the palliative care unit. The affected patients with palliative care needs then additionally fell under the responsibility of inpatient generalist palliative care, which was already heavily burdened by dying patients with COVID-19.

Although the implemented hygiene measures could be understood, some participants emphasized that they still need to be proportional to the circumstances.

"Why is it possible to provide pastoral care for a Methicillin-resistant Staphylococcus aureus patient [...] and NOT for a COVID-patient? In my opinion, everything is possible as usual with appropriate care and hygienic measures."

### Interdisciplinary and interprofessional cooperation

Nursing shortages already existing prior to the pandemic often caused insufficient interprofessional collaboration and arrangements. The SARS-CoV-2 pandemic exacerbated this shortage by adding time-consuming hygiene measures, elaborate visiting arrangements, and abscences of staff related to infection or quarantine. However, all participants also reported positive experiences: cohesion, solidarity and help were described, especially in the intensive care units.

“And what I found very POSITIVE was this good cooperation among the work team, how we all developed together.”

“that she/ I, for example, provided the patients with basic care together with the doctor, while the intensive care nurse also took care of the ventilation parameters. That was a really good Team work.”

In the meantime, the possibilities of interdisciplinary and interprofessional cooperation were also used beyond the hospital boundaries. Regarding seriously ill or dying patients, some participants reported a very helpful and close cooperation with the inpatient specialized palliative care.

"Well, I also remember caring for somebody at the end of life. Also [...] in collaboration with [...] the colleagues from palliative medicine. [...] I like to remember how we were able to accompany the relatives."

“What I would like to see in the future is that somehow palliative medicine and intensive care [...] get on the same page and at least once a week the patient's status is roughly [...] checked and potential [...] palliative medicine connections could take place."

### Determination of patient will

It was observed consistently that patients were treated in intensive care without discussing therapy goals beforehand. Overtreatment was a common result, further exacerbating resource constraints. Although this is not a new issue and was described by participants as a problem that existed before the pandemic, the pandemic exacerbated it [13]. During the focus groups, especially nurses controversially discussed the topic “goals of care”. Some participants explained that, despite clearly expressed patient wishes, timely changes in goals of care did not always occur. Thus, changes in therapy goals or limitations were hardly ever discussed.

"In the beginning, we also waited [...] with [...] a DNR [do not resuscitate], DNI [do not intubate] status".

Furthermore, within the focus groups, cases were described in which the patient's will was not obtained until a significant deterioration in their health status had occurred.

"They wait until it really gets worse and then ask for an advance directive, which is sometimes difficult for us especially in [...] oncology."

However, other participants had the impression that the open and detailed discussion of goals of care had increased. In this regard, limited resources provided a critical incentive to learn about patients' wishes at an earlier stage.

"We discussed goals of care A LOT and extensively. Therapy OUTCOMES were discussed [...] in MY opinion, perhaps even more than we used to."

### Possibility of saying goodbye

According to many participans, the lack of opportunities to say goodbye led to great distress of patients and their relatives, but also of the treatment team. Saying goodbye is an important part of relationships for all concerned. During the first wave, it was hardly possible to say goodbye to a patient with COVID-19. Some participants explicitly emphasized that farewells and support must also be possible during a pandemic.

"I BELIEVE it is IMPORTANT that people need to be accompanied [...] when they die."

Some participants described their experiences with patients in the dying process who could not be accompanied by their relatives because of their infection.

"So when COVID-patients passed away, unfortunately relatives could not join them. They passed away alone in that room, cared for by us."

In these cases, a (video)-telephone farewell was sometimes possible before intubation.

"But it could be quickly established that [...]video telephony [...] has become POSSIBLE, and that has actually been the case in SOME cases that patients, BEFORE they were intubated, could again actually [...] have contact with their relatives via video."

In the further course after the first wave, farewells were allowed again in many places. Individual participants reported that until the day of the interview, still no personal farewells were possible for infected patients.

"In the COVID-rooms they are still not allowed to go in. So that's still, if somebody dies there, the relatives cannot go to see their loved ones."

Participants described alternatives used in their clinic or ward. These include photos taken of the deceased to share with loved ones or a farewell room where deceased persons were laid out and given a farewell by the staff. Another participant reported a laying out room in the pathology department exclusively for deceased persons with infect, to provide an opportunity to say goodbye at least after death.

## Discussion

The results of this qualitative study demonstrate the hardships and burdens of inpatient generalist palliative care teams and thus emphasise the importance of special care for seriously ill and dying, even in pandemic times. As intensive care resources had been the focus of attention especially at the beginning, the importance of palliative care for seriously ill patients with COVID-19 and their relatives increased as the pandemic progressed. By June 2021, nearly 3.8 million people in Germany had become infected and more than 90,000 people had died in connection with the virus [14]. Furthermore, during the pandemic, patients who did not contract COVID-19 experienced severe courses of disease and died. In both cases, relatives were often denied the opportunity to say goodbye during lifetime or even after death. Therefore, inpatient generalist palliative care plays an important role in ensuring appropriate care for seriously ill and dying, even during a pandemic.

### Visiting regulations

According to the interviews, visiting bans and restrictions represent a particularly significant aspect limiting the quality of life of those affected. These precautionary measures are intended to counteract the further uncontrolled spread of the virus. Similar measures are being implemented in countries and hospitals around the world. Nevertheless, it is becoming increasingly clear that visits by patients' relatives are of great importance for their sense of well-being and the quality of care, especially for seriously ill and dying patients [15, 16]. Even after death, the possibility for bereaved relatives to say goodbye is a protective factor to avoid far-reaching consequences such as anxiety or depression due to disabled farewell and mourning processes [16, 17].

### Communication with relatives

In all focus groups, communication with relatives was presented as one of the most demanding challenge. The transmission of information by means of (video) telephone calls must be carefully prepared, ideally scheduled and subsequently documented. Time for questions and pauses should be implemented to an even greater extent than usual to be able to understand and process bad news. Furthermore, emotions and compassion should be verbalized more strongly, since the non-verbal part of communication is partially or completely omitted by the (video)telephony [18, 19]. The implementation and realization of clear communication concepts is also important in connection with prioritization decisions and to avoid overtherapy [19, 20]. If scarce resources lead to prioritize decisions, a feeling of withholding care can arise, which can only be circumvented through comprehensible communication, for example, according to the "SHARE talking map" (specifically to explain resource allocation), as illustrated in Table 4 [21].

**Table 4:** SHARE talking map

SHARE talking map
S how the guideline
H eadline what it means for the patient’s care
A ffirm the care you will provide
R espond to emotions
E mphasize that the same rules apply to everyone

If available, the specialized palliative care team can support the professionals of inpatient generalist palliative care in the communication with the relatives [22, 23].

### Hygiene measures

Adherence to hygiene measures is important to contain the pandemic. Therefore, these have a high priority in the care of seriously ill and dying patients, both to protect patients and staff. Material shortages, lack of training on correct use, and staff infection were reasons for uncertainty. An adequate supply of protective equipment is therefore most important for preventing transmission, and for maintaining visiting opportunities for relatives [24, 25]. In addition to doctors and nurses, other professions such as spiritual care, psychology or voluntary work also depend on the availability of protective equipment. Additional hygiene measures and the necessary training measures inevitably go hand in hand with a higher (time) expenditure, which further increases the shortage of personnel resources and thus the need for personnel.

### Interdisciplinary and interprofessional cooperation

Effective interdisciplinary and interprofessional collaboration characterized by trust is particularly helpful in pandemic times. The interview participants benefited from the support and knowledge they experienced through collaboration with staff in specialized palliative care. In this context, the importance of further training for nurses was particularly emphasized. Nurses have the closest contact to patients and thus very accurately perceive symptom burden and needs, so that knowledge, skills and attitudes acquired through training and closer collaboration can be directly applied. "Palliative Care Pandemic Packs", which are equipped with pocket cards and symptom-relieving medications, help to act more safely in the event of a large number of dying patients [26].

### Determination of patient will

The topic of respecting the patient's will was discussed intensively in all focus groups. Early definition of realistic goals of care while respecting the patient's will is particularly important. "Informed consent" decisions with the patient and, if necessary, with the patient's authorized family members/caregivers are requested [27]. Thereby ambiguity, uncertainty, and fear among patients and family members can be reduced, as well as among the therapeutic teams. The exceptional situation of a pandemic can lead to employees having to act outside their knowledge and area of competence more frequently, which can lead to the initiation of extensive therapies due to uncertainty [28, 29]. Here, the early involvement of specialized palliative care or ethical expertise can reduce the risk of overtherapy at the end of life [30]. This also applies to emergency departments or intensive care units, not least by providing support for Advance Care Planning (ACP) and initiating regular discussions of therapeutic goals [31, 32]. Here, too, it is advisable to involve nurses proactively in decision-making, because of their closer contact to the patients [32, 33].

### Possibility of saying goodbye

Saying goodbye at the deathbed has changed during the pandemic. If it is not possible to say goodbye in person, it is important for hospital staff to provide intensive support for digitally assisted farewells [34, 35]. Although the new possibilities of digitally assisted encounters and communication offer valuable opportunities, there are also risks and problems to be considered [36]. Communication in this context lacks a connection to the body, where the experience of death can be experienced through touch. Accordingly, some authors recommend good preparation and follow-up of communication in the digital-based setting. Tools have already been developed for this purpose (e.g. Goodbye Tool) [37].

Digital communication is not feasible for all patients or relatives. In the case of advanced age and dementia-related changes, e.g. the handling and comprehension of modern means of communication such as smartphones or tablets often represent a struggle [38]. From the perspective of the relatives, it is clear that the missing possibility to say goodbye can lead to an increased risk of complicated grief. When death occurs under difficult circumstances, grieving and coming to terms with it can be significantly more difficult for those affected [38]. Compassionate, proactive communication is paramount. Furthermore, a face-to-face meeting between family members and the staff who cared for the deceased can be important to answer questions. This is also helpful at a distance from the acute event.

## Limitations

To comply with the Data Protection Act only very little demographic data could be collected. Furthermore, the interviewed groups were not homogeneous due to interprofessionalism and interdisciplinarity. In addition, it is noticeable here that the number of physician participants is rather high in contrast to the nurses, although the nurses tend to be in the majority in everyday hospital life. Whether this is due to a current shortage of nursing staff in Germany can only be assumed. We did not query specialized palliative care contents as we wanted the most open discussion possible to emerge. The chosen method intended to evolve topics within the group and external influences were only given if the discussions came to a standstill. With regards to data saturation another interview could have validated the saturation if there would have been more time at hand. Furthermore, there has been no member check of the transcripts due to the limited amount of time.

In some circumstances, the limitations stated may have impacted the findings, i.g. selection bias or interviewer bias. Furthermore, the qualitative results of our study need to be further investigated within quantitative study designs.

## Conclusion

Continuing visits to critically ill and dying patients is considered important and should be maintained in pandemic times if whenever possible. Hygiene concepts and the necessary materials in sufficient quantities are a prerequisite for this. Communication via alternative channels proved to be helpful for all participants. Based on those findings, a quantitative survey is currently in progress.

## Data Availability

All data generated or analysed during this study are included in this published article and its supplementary information files.

## References

[1] Robert-Koch-Institut. RKI COVID-19 Germany. 2021. https://experience.arcgis.com/experience/478220a4c454480e823b17327b2bf1d4. Accessed 15 Apr 2021.

[2] Deutsche Interdisziplinäre Vereinigung für Intensiv- und Notfallmedizin (DIVI) e.V. DIVI Intensivregister. 2021. https://www.intensivregister.de/#/aktuelle-lage/zeitreihen. Accessed 15 Apr 2021.

[3] Ramshorn-Zimmer A, Fakler J, Schröder R, Stöhr R, Kohls E, Gries A (2020). Notaufnahme während der Corona Pandemie: Weniger Non-Covid-19-Notfälle. Deutsches Ärzteblatt.

[4] Stock L, Brown M, Bradley G (2020). First do no harm with COVID-19: corona collateral damage syndrome. West J Emerg Med.

[5] Deutschen Interdisziplinären Vereinigung für Intensiv- und Notfallmedizin (DIVI), Deutschen Gesellschaft für Interdisziplinäre Notfall- und Akutmedizin (DGINA), Deutschen Gesellschaft für Anästhesiologie und Intensivmedizin (DGAI), Deutschen Gesellschaft für Internistische Intensivmedizin und Notfallmedizin (DGIIN), Deutschen Gesellschaft für Neurointensiv- und Notfallmedizin (DGNI), Deutschen Gesellschaft für Pneumologie und Beatmungsmedizin (DGP), Deutschen Gesellschaft für Palliativmedizin (DGP), Akademie für Ethik in der Medizin. Entscheidungen über die Zuteilung intensivmedizinischer Ressourcen im Kontext der COVID-19-Pandemie. 2020.

[6] Nehls W, Delis S, Haberland B, Maier BO, Sänger K, Tessmer G, et al. Therapie von PatientInnen mit COVID-19. [Management of Patients with COVID-19 - Recommendations from a Palliative Care Perspective]. Pneumologie. 2020;74:652–9. doi:10.1055/a-1156-2759. 10.1055/a-1156-2759 PMC7645808 32316056

[7] Quill TE, Abernethy AP (2013). Generalist plus specialist palliative care–creating a more sustainable model. N Engl J Med.

[8] World Health Organization. Pandemic preparedness. 12.01.2021. https://www.euro.who.int/en/health-topics/communicable-diseases/influenza/pandemic-influenza/pandemic-preparedness. Accessed 15 Jan 2021.

[9] Bausewein C, Simon S. Nationale Strategie für die Betreuung von schwerkranken und sterbenden Menschen und ihren Angehörigen in Pandemiezeiten (PallPan). 2021. https://zenodo.org/record/5012504. Accessed 6 Jul 2021.

[10] Tong A, Sainsbury P, Craig J (2007). Consolidated criteria for reporting qualitative research (COREQ): a 32-item checklist for interviews and focus groups. Int J Qual Health Care.

[11] Dresing T, Pehl T (2018). Praxisbuch Transkription: Regelsysteme, Software und praktische Anleitungen für qualitative ForscherInnen.

[12] Kuckartz U (2016). Qualitative Inhaltsanalyse: Methoden, Praxis, Computerunterstützung.

[13] You JJ (2015). Barriers to goals of care discussions with seriously ill hospitalized patients and their families: a multicenter survey of clinicians. JAMA Intern Med.

[14] Robert-Koch-Institut. RKI COVID-19 Germany Dashboard. 2021. https://experience.arcgis.com/experience/478220a4c454480e823b17327b2bf1d4. Accessed 3 Mar 2021.

[15] Ministerium für Arbeit, Gesundheit und Soziales des Landes Nordrhein-Westfalen. Verordnung zum Schutz vor Neuinfizierungen mit dem Coronavirus SARS-CoV-2(Coronaschutzverordnung – CoronaSchVO) Vom 5.März 2021; 2021.

[16] Münch U, Müller H, Deffner T, Schmude A von, Kern M, Kiepke-Ziemes S, Radbruch L. Empfehlungen zur Unterstützung von belasteten, schwerstkranken, sterbenden und trauernden Menschen in der Corona-Pandemie aus palliativmedizinischer Perspektive : Empfehlungen der Deutschen Gesellschaft für Palliativmedizin (DGP), der Deutschen Interdisziplinären Vereinigung für Intensiv- und Notfallmedizin (DIVI), des Bundesverbands Trauerbegleitung (BVT), der Arbeitsgemeinschaft für Psychoonkologie in der Deutschen Krebsgesellschaft, der Deutschen Vereinigung für Soziale Arbeit im Gesundheitswesen (DVSG) und der Deutschen Gesellschaft für Systemische Therapie, Beratung und Familientherapie (DGSF). [Recommendations for the support of suffering, severely ill, dying or grieving persons in the corona pandemic from a palliative care perspective : Recommendations of the German Society for Palliative Medicine (DGP), the German Interdisciplinary Association for Intensive and Emergency Medicine (DIVI), the Federal Association for Grief Counseling (BVT), the Working Group for Psycho-oncology in the German Cancer Society, the German Association for Social Work in the Healthcare System (DVSG) and the German Association for Systemic Therapy, Counseling and Family Therapy (DGSF)]. Schmerz. 2020;34:303–13. doi:10.1007/s00482-020-00483-9. 10.1007/s00482-020-00483-9 PMC7265165 32488422

[17] Wallace CL, Wladkowski SP, Gibson A, White P. Grief during the COVID-19 pandemic: considerations for palliative care providers. J Pain Symptom Manage. 2020:e70-e76. doi:10.1016/j.jpainsymman.2020.04.012. 10.1016/j.jpainsymman.2020.04.012 PMC7153515 32298748

[18] Mistraletti G, Gristina G, Mascarin S, Iacobone E, Giubbilo I, Bonfanti S (2020). How to communicate with families living in complete isolation. BMJ Support Palliat Care.

[19] Chua IS, Jackson V, Kamdar M (2020). Webside manner during the COVID-19 pandemic: maintaining human connection during virtual visits. J Palliat Med.

[20] Anantham D, Chai-Lim C, Zhou JX, Phua GC (2020). Operationalization of critical care triage during a pandemic surge using protocolized communication and integrated supportive care. J Intensive Care.

[21] Back A, Tulsky JA, Arnold RM (2020). Communication skills in the age of COVID-19. Ann Intern Med.

[22] Sheehan J, Ho KS, Poon J, Sarosky K, Fung JY (2020). Palliative care in critically ill COVID-19 patients: the early New York City experience. BMJ Support Palliat Care.

[23] Lopez S, Finuf KD, Marziliano A, Sinvani L, Burns EA (2021). Palliative care consultation in hospitalized patients With COVID-19: a retrospective study of characteristics, outcomes, and unmet needs. J Pain Symptom Manage.

[24] Downar J, Seccareccia D (2010). Palliating a pandemic: "all patients must be cared for". J Pain Symptom Manage.

[25] Arya A, Buchman S, Gagnon B, Downar J (2020). Pandemic palliative care: beyond ventilators and saving lives. CMAJ.

[26] Ferguson L, Barham D (2020). Palliative care pandemic pack: a specialist palliative care service response to planning the COVID-19 pandemic. J Pain Symptom Manage.

[27] Deutsche Krebsgesellschaft, Deutsche Krebshilfe, AWMF. Leitlinienprogramm Onkologie: Palliativmedizin: Palliativmedizin für Patienten mit einer nicht-heilbaren Krebserkrankung, Lang-version 2.0, 2019, AWMF-Registernummer: 128/001OL. 17.05.2021. https://www.leitlinienprogramm-onkologie.de/leitlinien/palliativmedizin/. Accessed 17 May 2021.

[28] Simpkin AL, Schwartzstein RM (2016). Tolerating uncertainty - the next medical revolution?. N Engl J Med.

[29] Koffman J, Gross J, Etkind SN, Selman L (2020). Uncertainty and COVID-19: how are we to respond?. J R Soc Med.

[30] Lee J, Abrukin L, Flores S, Gavin N, Romney M-L, Blinderman CD, Nakagawa S (2020). Early intervention of palliative care in the emergency department during the COVID-19 pandemic. JAMA Intern Med.

[31] Schoenherr LA, Cook A, Peck S, Humphreys J, Goto Y, Saks NT (2020). Proactive identification of palliative care needs among patients with COVID-19 in the ICU. J Pain Symptom Manage.

[32] Adams JA, Bailey DE, Anderson RA, Docherty SL (2011). Nursing roles and strategies in end-of-life decision making in acute care: a systematic review of the literature. Nurs Res Pract.

[33] Raftery C, Lewis E, Cardona M (2020). The crucial role of nurses and social workers in initiating end-of-life communication to reduce overtreatment in the midst of the COVID-19 pandemic. Gerontology.

[34] Selman LE, Chao D, Sowden R, Marshall S, Chamberlain C, Koffman J (2020). Bereavement support on the frontline of COVID-19: recommendations for hospital clinicians. J Pain Symptom Manage.

[35] Chidiac C, Feuer D, Naismith J, Flatley M, Preston N. Emergency palliative care planning and support in a COVID-19 pandemic. J Palliat Med. 2020:752–3. doi:10.1089/jpm.2020.0195. 10.1089/jpm.2020.0195 32311287

[36] Fusi-Schmidhauser T, Preston NJ, Keller N, Gamondi C (2020). Conservative management of COVID-19 patients-emergency palliative care in action. J Pain Symptom Manage.

[37] Frydman JL, Choi EW, Lindenberger EC (2020). Families of COVID-19 patients say goodbye on video: a structured approach to virtual end-of-life conversations. J Palliat Med.

[38] Moore KJ, Sampson EL, Kupeli N, Davies N. Supporting families in end-of-life care and bereavement in the COVID-19 era. Int Psychogeriatr. 2020:1245–8. doi:10.1017/S1041610220000745. 10.1017/S1041610220000745 PMC7235296 32349850

